# Genome-Wide Identification of PLATZ Transcription Factors in *Ginkgo biloba* L. and Their Expression Characteristics During Seed Development

**DOI:** 10.3389/fpls.2022.946194

**Published:** 2022-06-23

**Authors:** Xin Han, Hao Rong, Yating Tian, Yanshu Qu, Meng Xu, Li-an Xu

**Affiliations:** Key Laboratory of Forestry Genetics and Biotechnology of Ministry of Education, Co-Innovation Center for Sustainable Forestry in Southern China, Nanjing Forestry University, Nanjing, China

**Keywords:** *Ginkgo biloba* L., PLATZ, seed development, expression pattern, subcellular localization

## Abstract

Plant AT-rich protein and zinc-binding protein (PLATZ) is a class of plant-specific zinc-dependent DNA-binding protein that binds to A/T-rich DNA sequences. PLATZ plays an important role in seed development, water tolerance, and cell proliferation in early plant growth. In this study, 11 *GbPLATZ*s were identified from the ginkgo genome with complete PLATZ-conserved domains, which represents a smaller number compared with angiosperms. Multi-species phylogenetic analysis showed that PLATZ genes were conserved in seed plants, and the 11 members were represented by four groups, among which groups I and II were closely related. Analysis of gene structures, sequence module characteristics, and expression patterns showed that *GbPLATZ*s were similar within and differed between groups. RNA-seq and qRT-PCR results showed that *GbPLATZ*s had distinct expression patterns. Most genes were associated with seed development, among which six genes were highly related. Subcellular localization experiments showed that six GbPLATZ proteins related to seed development were localized in the nucleus, suggesting that they might function as traditional transcription factors. This study provides a basis for understanding the structural differentiation, evolutionary characteristics, expression profile, and potential functions of PLATZ transcription factors in *Ginkgo biloba*.

## Introduction

The growing wealth of genomic data from an increasingly diverse set of taxa provides the unprecedented potential to elucidate the genome biology and evolution of land plants (Marks et al., 2021). The study of gene families has become an essential means to analyze the function, structure, and evolutionary characteristics of genes. DNA-binding transcription factors (TFs), the products of some gene families, positively or negatively regulate gene expression in response to developmental and environmental changes by binding to the cis-acting elements in upstream promoter regions (Fu et al., 2020). The plant AT-rich sequences and zinc-binding proteins (PLATZ) TF family is endemic to plants and was first isolated from peas (Yukio et al., 2001). PLATZ has two distant zinc-binding regions which are required for DNA binding, C-x_2_-H-x_11_-C-x_2_-C-x_(4–5)_-Cx_2_-C-x_(3–7)_-H-x_2_-H and C-x_2_-C-x_(10–11)_-C-x_3_-C.

PLATZ TFs play an important role in seed development, except for the model plant Arabidopsis, previous research has mostly focused on food crops. The pea PLATZ1 non-specifically binds to A/T-rich sequences and represses the transcription of the pea GTPase *pra2* and plastocyanin *petE* genes (Yukio et al., 2001). *AtPLATZ1* (ABA-INDUCED expression 1) is involved in ABA-inhibited primary root elongation *via* modulation of ROS homeostasis in Arabidopsis (Dong et al., 2021). *AtPLATZ2* negatively regulates salt tolerance in Arabidopsis seedlings by directly suppressing the expression of the *CBL4/SOS3* and *CBL10/SCaBP8* genes. (Liu et al., 2020). *AtPLATZ3* (ORESARA15) enhances leaf growth by promoting the rate and duration of cell proliferation in the early growth stages and suppresses leaf senescence in later stages (Kim et al., 2018; Jun et al., 2020). *AtPLATZ7* (*RITF1*, RGF1-INDUCIBLE TRANSCRIPTION FACTOR1) plays a central role in mediating *RGF1* signaling, which controls root meristem size through ROS signaling (Yamada et al., 2020). In rice, two PLATZ TFs related to grain size, *GL6* and *SG6*, were identified by QTL mapping and mutant analysis, respectively. *GL6* (Os06g0666100) participates in RNAPIII transcription machinery by interacting with RPC53 and TFC1 to promote cell proliferation, which positively controls grain length (Wang et al., 2019). *SG6* is preferentially expressed in panicles and determines grain size by regulating spikelet hull cell division (Zhou and Xue, 2020). *ZmPLATZ2* (GRMZM2G311656) binds to the CAA AAA AA element in the *ZmSSI* promoter and mediates the Glu signal pathway to positively regulate starch synthesis in maize (Li et al., 2021). *ZmPLATZ12* (GRMZM2G006585, *Floury3*, *FL3*) is regulated by genomic imprinting and encodes for a PLATZ protein that interacts with RNAPIII in the biogenesis of tRNA and 5S rRNA, which may affect endosperm development and storage reserve maintenance (Li et al., 2017). *GmPLATZ1* is a PLATZ TF induced in soybean (*Glycine max* L.)by abiotic stress, including drought, high salinity, and abscisic acid (ABA), and is implicated in developmental processes, such as germination (So et al., 2015). Transgenic Arabidopsis, ectopically expressing the cotton PLATZ TF *GhPLATZ1*, exhibited faster seed germination and higher seedling establishment under salt and mannitol stress than those of wild-type seeds (WT). This enhanced osmotic insensitivity suggests that *GhPLATZ1* may regulate hormone-mediated osmotic stress during cotton seed germination and seedling establishment (Zhang et al., 2018). Indeed, PLATZ TFs play both direct and indirect roles, and both positive and negative regulatory roles, which reflect the complexity of their functions at the level of transcriptional regulation.

*Ginkgo biloba* L. is the only existing Ginkgopsida species of gymnosperms. It has a critical evolutionary status in seed plants as it is considered to be the link between angiosperms and cryptogams (Wu et al., 2018). In contrast to the double fertilization of angiosperms, the endosperm of gymnosperm seeds develops from haploid functional megaspores (Linkies et al., 2010). Studies have shown that the development of the ginkgo embryo lags behind the formation of its endosperm. After pollination, ginkgo pollen remains dormant for 4–5 months in the storage chamber, during which time the three layers of the seed coat and endosperm rapidly differentiate and proliferate (Wang et al., 2006, 2009). After harvest, ginkgo seeds need to undergo dormancy before germinating, which belongs to the ancestral morphophysiological dormancy type (Jia et al., 2020). Ginkgo also has economic value. *Ginkgo biloba* extract (GBE) is a popular health product, and therapeutic activity has been reported for the ginkgo flavone, ginkgolide, and bilobalide (Van Beek and Montoro, 2009; Hua et al., 2017). The ginkgo nut is the biggest edible seed in gymnosperms and has a rich starchy endosperm but with some allergenicity (Sado et al., 2019).

High-quality ginkgo genomes sequences have recently been assembled (Liu et al., 2021b), and some full-length transcriptome data have been sequenced and annotated by different research groups (Sun et al., 2020; Han et al., 2021), which greatly enriches the available resources for gene family analysis. Studies have shown that genome replication events often lead to an increase in gene family members and redundancy of functions. Ginkgo only experienced one replication event common to the ancestors of seed plants (~320 Mya; Alix et al., 2017; Niu et al., 2021). Considering the information in the iTAK database (Zheng et al., 2016), the ginkgo TF family generally has fewer members. As a result, studies on the functions of the family members tend to be representative of the species. As a plant-specific gene associated with seed development, there is no related research on the PLATZ TF in gymnosperms. Therefore, genome-wide identification and analysis of PLATZ in ginkgo could provide valuable insights into the biology and evolution of the species.

The identification of the structure and function of PLATZ TFs in ginkgo could elucidate the variability of PLATZ members in the evolution of seed plants, especially gymnosperms. In this study, we describe ginkgo PLATZs according to the following aspects: (1) genome-wide identification of *GbPLATZ* genes based on the latest genome data; (2) PLATZ phylogeny and evolution of ginkgo, Norway spruce, Arabidopsis, and maize; (3) identification of chromosome distribution and gene replication; (4) analysis of conserved motifs and cis-elements of *GbPLATZ*s; (5) prediction of the physicochemical properties and structure of GbPLATZ proteins; (6) *GbPLATZ* expression profile analysis; and (7) subcellular localization prediction and partial validation of *GbPLATZ*s.

## Materials and Methods

### Plant Material and RNA Extraction

We collected root, stem, and leaf tissue from 3-month-old ginkgo seedlings. The male/female bud, as well as leaves and seeds at different developmental stages, were obtained from two adult trees at Nanjing Forestry University (Nanjing, China) in 2020. The kernels were obtained by splitting the middle coat of ginkgo seeds and tearing off the inner coat. All plant materials were frozen by liquid nitrogen and stored at 80°C. FastPure Plant Total RNA Isolation Kit (Vazyme, Nanjing, China) was used for RNA extraction of all samples. RNA degradation and contamination were detected by 1.5% agarose gel. RNA purity and concentration were detected using a NanoPhotometer spectrophotometer (Implen, Westlake Village, CA, United States). Reverse transcription of RNA was performed using Takara PrimeScript RT Master Mix (Takara, Beijing, China).

### Identification of PLATZ Family Genes in Ginkgo

The identification of PLATZ gene family members in ginkgo includes three approaches. (1) Similar to the method described in other studies, gene family members were predicted according to conserved domains (Bi et al., 2016). Based on the newly published genome (Liu et al., 2021b) as well as protein sequence and annotation data, we used the hidden Markov model with parameters of PLATZ TF in the PFAM database (Finn et al., 2014; PF04640).^1^ Here, “hmmbuild” in the HMMER procedure set was used to model the Stockholm format file (SEED), and “hmmsearch” was used to identify the possible PLATZ genes in the whole-genome protein sequence file of ginkgo. The specific parameter was “-Z 61295632 -E 1000 -- cpu 4 HMM pfamseq” (Qu et al., 2019). (2) Local BlastP (Schaffer et al., 2001; e-value = 1e-5) was used to compare the Fasta-format (SEED) file with the whole-genome protein sequence file of ginkgo, and potential PLATZ genes were identified according to sequence similarity. (3) TFs predicted by the genome in the plant TF database iTAK (TF data of ginkgo in this database are based on another earlier version of the genome; Guan et al., 2016) were integrated with the PLATZ family members obtained in the previous two steps, and redundant was removed.

For each candidate PLATZ family member, we assessed the transcript and protein sequence of each member based on the full-length ginkgo transcript dataset, and the longest isoform was selected for downstream analysis. TBtools (Chen et al., 2020) was used to analyze the basic information of ginkgo genome chromosomes, and MG2C (Chao et al., 2021) was used to draw the chromosome location map according to the location of *GbPLATZ*s, which were named according to the chromosome order. TBtools was also used to visualize the SeqLogo of GbPLATZ amino acid sequences.

### Phylogenetic and Intraspecific Collinearity Analysis of *GbPLATZ*s

Using MEGA X (Kumar et al., 2018), ClustalW was used to compare the amino acid sequences of 11 *GbPLATZ*s; a maximum likelihood phylogenetic tree was constructed using the Jones-Taylor-Thornton (JTT) model; and a phylogenetic test was conducted using a bootstrap method with 1,000 iterations. In a similar manner, we compared PLATZ family members of multiple species and constructed phylogenetic trees based on protein sequences. Intraspecific collinearity analysis was performed using the “MCScanX” plug-in of TBtools, based on the ginkgo genome, annotated files, and amino acid sequences of *GbPLATZ*s.

### Protein Property Analysis

ExPASy online resources^2^ were used to predict the physical and chemical parameters of the ginkgo PLATZ protein, including molecular weight (MW), theoretical isoelectric point (PІ), grand average hydropathicity (GRAVY), instability index, and aliphatic index (Gasteiger et al., 2003). The SOPMA server (Geourjon and Deleage, 1995) was used to predict the secondary structure of protein sequences for the 11 *GbPLATZ*s and obtain the proportion of various secondary structures. AlphaFold2 (Jumper et al., 2021) was used to predict the tertiary structure of proteins through Google Colaboratory (Carneiro et al., 2018), and Chimera X (v 1.3; Pettersen et al., 2021) was used to visualize the results.

### Expression Profile of *GbPLATZ*s

A total of 17 groups of RNA-seq data from five projects were used to analyze the gene expression pattern of *GbPLATZ*s; developmental stage data of seeds and leaves are based on the previous sequencing data from our laboratory (Han et al., 2021; Liu et al., 2021a). Data for the remaining three projects were downloaded from the NCBI database (Supplementary Table 1), including roots, stems, leaves, flowers, seedlings, and seeds of ginkgo. The “aln” function of BWA v-0.7.5(Li and Durbin, 2009) was accessed using our own script to calculate the expression amount with CDS as the target. The read_num of each gene was obtained using the “view” and “idxstats” functions of Samtools (Li et al., 2009). In this study, TPM (transcripts per kilobase of exon model per million mapped reads) values represent relative gene expression and are used to facilitate comparison between tissues. The expression spectrum of *GbPLATZ*s was visualized by TBtools.

The 11 ginkgo genes were quantified by real-time quantification PCR (qRT-PCR) in roots, stems, leaves, flowers, kernels, and seed coats, with at least three biological replicates. Gene specific primers were designed using Beacon Designer.^3^ The housekeeping genes *GbGAPDH* and *GbEIF3D* were used as endogenous references (all primers are shown in Supplementary Table 2). The qRT-PCR was performed with SYBR Premix Ex Taq (Takara, Tokyo, Japan) and a ViiA 7 Real-Time PCR System (Thermo Fisher Scientific, Waltham, MA, United States), with three technical replicates for each reaction. PCR amplification was performed under the following conditions: 95°C for 30 s; 95°C for 5 s, 60°C for 30 s, and 72°C for 15 s with 40 cycles; 95°C for 10 s. Relative expression levels were calculated using 2^–ΔΔCt^. Data analysis and visualization were performed using GraphPad Prism v 8.0.0 for Windows (GraphPad, San Diego, CA, United States).

### Prediction and Validation of GbPLATZ Subcellular Localization

Plant-mPloc (Chou and Shen, 2010) was used to predict the subcellular localization for amino acid sequences of the 11 *GbPLATZ*s. The sequence predicted with a nuclear localization was submitted to Identification of Nucleus Signal Peptide from Protein Primary Sequence (INSP; Guo et al., 2020) for nuclear localization signal recognition. To verify the prediction results, six *GbPLATZ*s expressed during ginkgo seed development were tested by protoplast subcellular localization. The CDS of these genes without termination codons were constructed into the pNC-AMP-GFP-C/N vector (Yan et al., 2020), to obtain *35S::GFP-GbPLATZ2/4/7/8/9/10* and *35S::GbPLATZ2/4/7/8/9/10-GFP*. Transient transformation of ginkgo protoplasts mediated by PEG was based on a method optimized by our laboratory (unpublished data). High-activity protoplasts were extracted by enzymatic hydrolysis from the leaves of ginkgo seedlings. Protoplasts and high-quality plasmids were treated with PEG4000 solution for 15 min, for transformation, and incubated for 14 h before observation. Each transient transformation experiment was repeated at least three times. GFP fluorescence was recorded using a fluorescence microscope (Scope A1 Carl Zeiss, Jena, Germany).

## Result

### PLATZ Transcription Factors in Ginkgo

**Figure 1:**
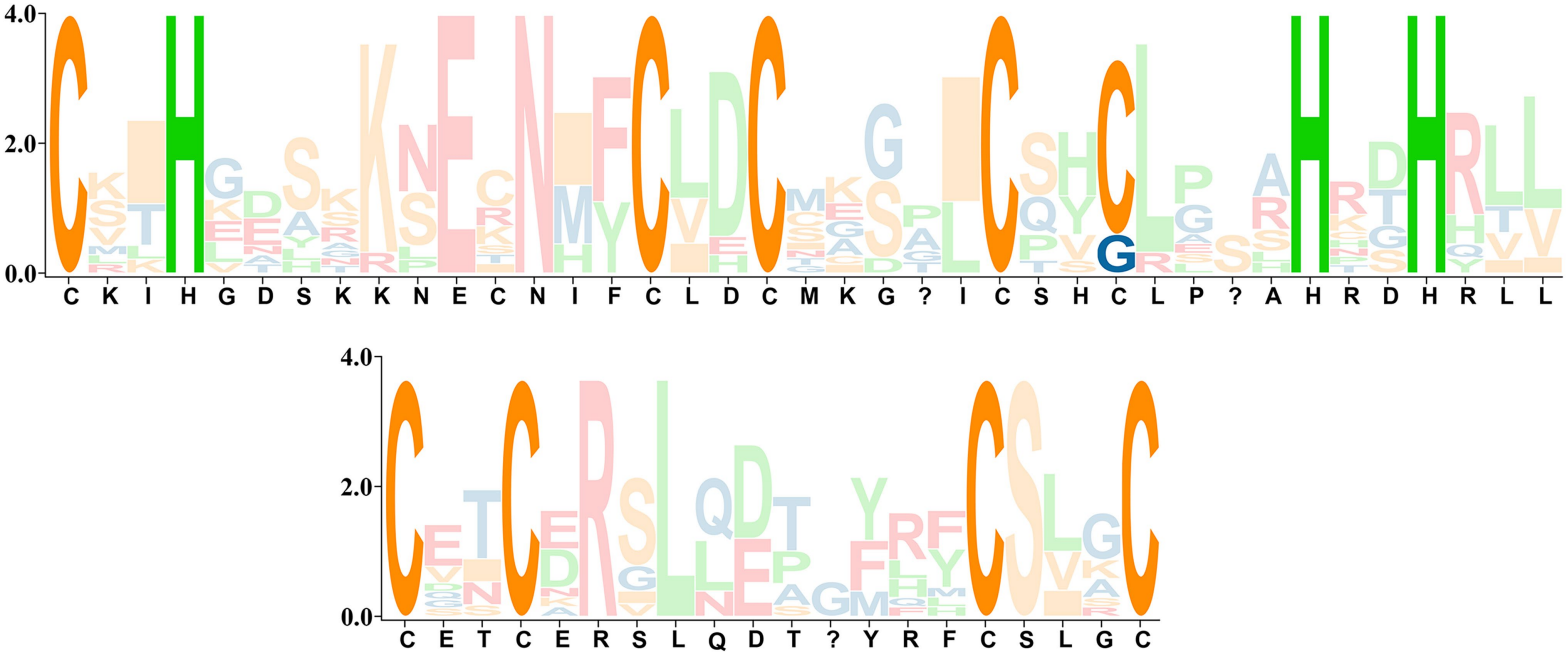
SeqLogo of two conserved regions of the PLATZ transcription factor in ginkgo.

According to the hidden Markov model (PF04640) and BlastP homology alignment of ginkgo proteins, a total of 11 candidate sequences were screened within the ginkgo genome (Supplementary Material). The sequence with an e-value nearest the threshold did not conform to the characteristics of the conserved domain for this TF family. Combined with the six PLATZs in iTAK database, Table 1 presents the structural information of the 11 *GbPLATZ*s after removing redundancy by sequence alignment. Through homologous sequence comparison using the Pfam, SMART (Letunic et al., 2021) and NCBI CCD databases, we confirmed that all candidate sequences contained the complete PLATZ domain. The number of PLATZ TFs in ginkgo was less than that reported for other species in systematic studies (Wang et al., 2018; Azim et al., 2020; Fu et al., 2020; Yang and Nie, 2021), and close to that of *Arabidopsis thaliana* (12; Lamesch et al., 2012). All 11 *GbPLATZ*s contained two unique conserved regions of PLATZ with cysteine and histidine at a certain distance apart, though *GbPLATZ1* and *GbPLATZ5* had cysteine replaced by glycine in Domain1 (Figure 1; Supplementary Figure 1). The 11 *GbPLATZ*s have lengths ranging from 693 bp to 1,047 bp. Except for *GbPLATZ1* and *GbPLATZ5*, which do not contain introns, the other genes have at least three introns. Except for *GbPLATZ5/8/10*, eight members are located on the justice chain of the genome.

**Table 1:** Eleven *GbPLATZs* identified from the complete genome of *Ginkgo biloba*.

Gene_name	Gene_id^a^	Gene_id^b^	ORF (bp)	Exons-introns	Chr_No.^b^	Chr_from	Chr_to	Strand
*GbPLATZ1*	Gb_39126^*^	chr2.201	1,047	1-0	2	73514318	73515363	+
*GbPLATZ2*	Gb_27083&Gb_27084	chr2.1059	756	4-3	2	381563586	381564732	+
*GbPLATZ3*	Gb_40617	chr3.742	693	4-3	3	199647484	199648612	+
*GbPLATZ4*	Gb_01585	chr3.2215	774	5-4	3	683595028	683597679	+
*GbPLATZ5*	Gb_32018^*^	chr3.2248	693	1-0	3	697889703	697890638	−
*GbPLATZ6*	Gb_20473&Gb_20459	chr6.1186	891	4-3	6	37019735	370198625	+
*GbPLATZ7*	Gb_01198^*^	Chr6.2014	753	5-4	6	638936540	638939023	+
*GbPLATZ8*	Gb_29488^*^	chr7.1438	762	5-4	7	463874992	463877882	−
*GbPLATZ9*	Gb_01089	chr9.1892	777	4-3	9	599898085	599899267	+
*GbPLATZ10*	Gb_13705^*^	chr10.1557	840	4-3	10	487043535	487045794	−
*GbPLATZ11*	Gb_33451^*^	chr10.1967	750	4-3	10	651307826	651308794	+

a The genome comes from Guan et al. (2016).

* Indicates that the Gene_id is in the iTAK database.

b The genome comes from Liu et al. (2021b), the chromosomal position information is based on it.

### Phylogenetic Analysis of *GbPLATZs*

**Figure 2:**
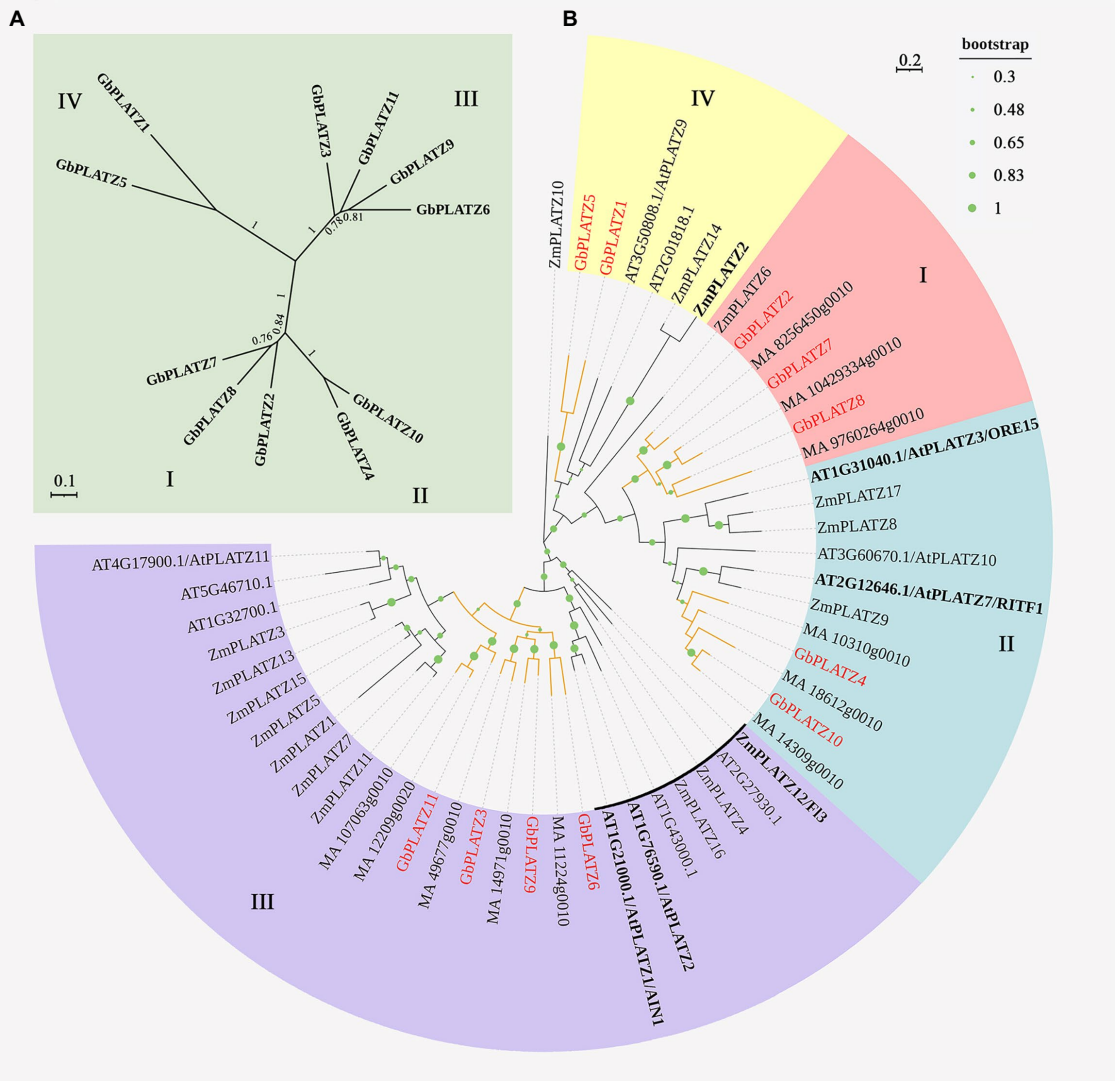
Phylogenetic analysis of the *GbPLATZ*s. **(A)** Maximum likelihood trees of 11 PLATZ transcription factors in *Ginkgo biloba*. I–IV represents four categories based on phylogenetic topology. **(B)** Maximum likelihood tree for ginkgo (Gb), Norway spruce (MA), maize (Zm), and Arabidopsis (AT) PLATZ TFs, where *GbPLATZ*s and their branches are represented in red. The gymnosperms branches are highlighted in brown. The branch length is proportional to the rate of amino acid change. The size of a point on a branch represents the bootstrap value of the branch.

To further analyze the genetic relationship and evolution of the *GbPLATZ*s, the 11 genes were compared with multiple sequences, and their relatedness was analyzed using a phylogenetic tree. Subsequently, a multi-species phylogenetic tree was constructed to clarify the classification and potential functions of PLATZ TFs in ginkgo compared to the seed crop maize, model plant Arabidopsis, and gymnosperm Norway spruce. From the topological structure of the two phylogenetic trees, *GbPLATZ*s can be divided into four groups (Figures 2A,B), each with at least two members (group III has four members, and group I has three). Among the four groups, groups I and II had the lowest genetic distance. In the multi-species phylogenetic analysis (Figure 2B), different groups of *GbPLATZ*s are distributed on different branches of the tree. Except for group IV, the *GbPLATZ*s of the other groups were closely related to the Norway spruce gene, which was highly conserved between PLATZ protein sequences of gymnosperms. The presence of group IV emphasizes ginkgo’s phylogenetic uniqueness. Several Arabidopsis and maize proteins were closely related to those of ginkgo group I and II, and two Arabidopsis proteins were involved in promoting cell proliferation. A relatively large number of maize genes occurred near the branches of ginkgo group III, which may indicate that maize, as a domesticated crop, has been enhanced in functions related to seed development. Some Arabidopsis and maize genes were relatively distant from gymnosperms, which may be a new group derived from angiosperms (branches marked by black arcs). Among these, two Arabidopsis genes in group III were associated with abiotic stress adaptation and maize *Fl3* was associated with endosperm development and storage accumulation. In group IV, only one branch consisting of two Arabidopsis and two maize genes converged; though with less than 0.5 support, it may not qualify as a group. Here, *ZmPLATZ2* has been associated with the forward synthesis of corn starch. Structure determines function, and similar genes may have similar functions; thus the grouping in the phylogenetic tree reflects the differences in gene function during evolution (He et al., 2021). Group IV showed low expression levels in all tissues and has a relatively weak function, which may be stimulated by some spatio-temporal specific tissue state or special stress not covered in this study.

### Chromosome Localization and Collinearity Analysis of *GbPLATZ*s

**Figure 3:**
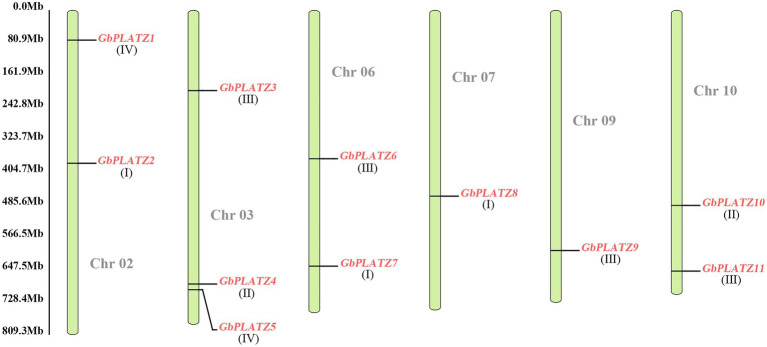
Mapping of *GbPLATZ*s gene in ginkgo chromosomes. The dark gray number represents the corresponding chromosome, the length of the green bar represents the length range of the chromosome, the scale on the left represents the distance on the chromosome position, and the 11 *GbPLATZ* genes are represented in red. The Roman letters under the gene name represent the group.

We assessed chromosome localization of *GbPLATZ*s based on ginkgo genome and structural annotation data (Figure 3). The 11 genes were unevenly distributed across six chromosomes (ginkgo had 12 chromosomes). The number of *GbPLATZ*s was the highest on chromosome 3, with three; chromosomes 2, 6, and 10 had two each; and chromosomes 7 and 9 had one each. Except for *GbPLATZ4* and *GbPLATZ5*, all other genes were scattered on the chromosomes with no apparent pattern in localization. No collinearity was observed among the 11 PLATZ TFs of *G. biloba*.

### Gene Structure and Conserved Domain Characteristics of *GbPLATZ*s

**Figure 4:**
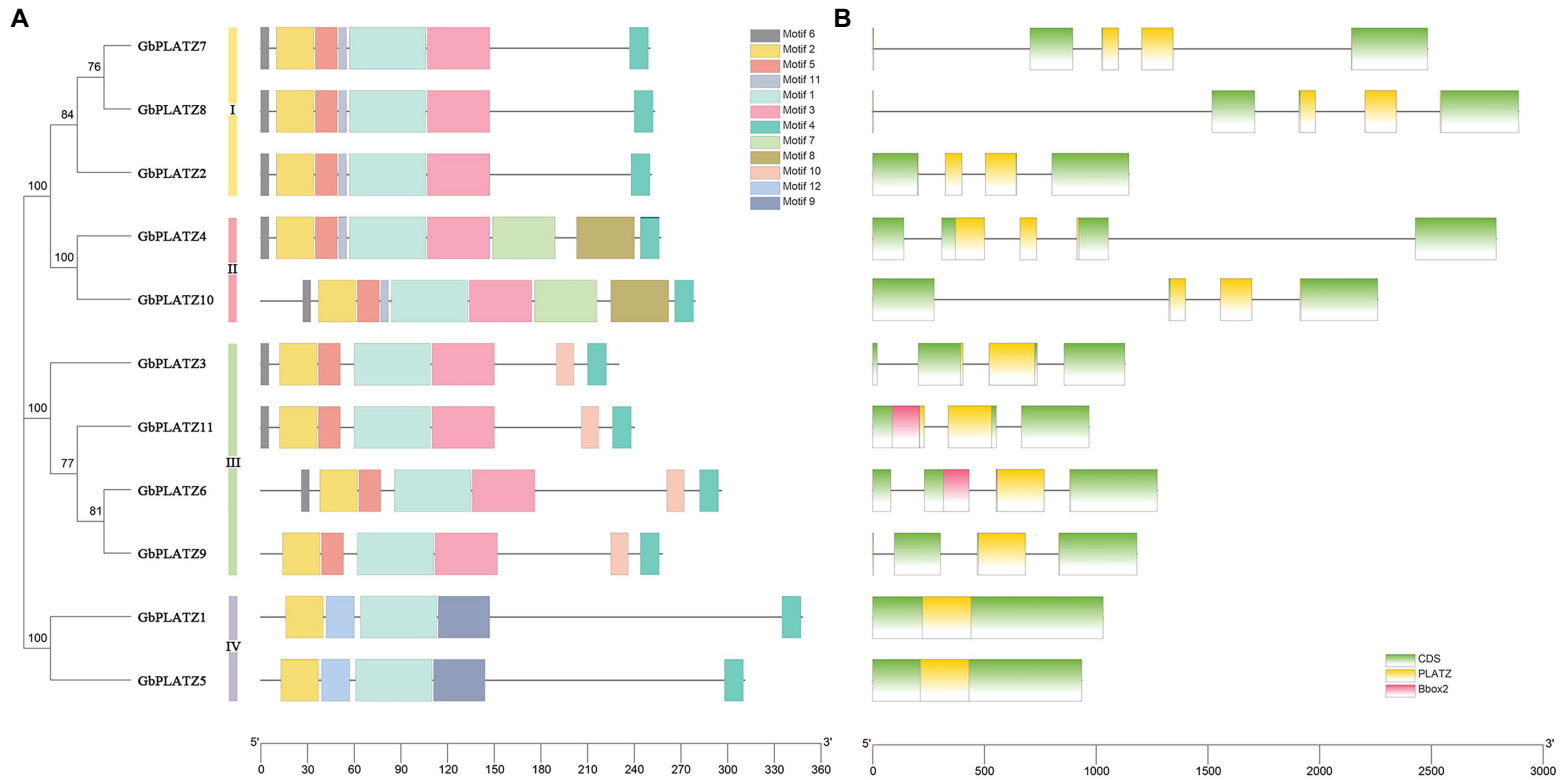
Motif structure, gene structure, and family conservation analysis of *GbPLATZ*s. **(A)** The 12 MEME motifs are similar within groups and different between groups. **(B)** According to genome annotation data, *GbPLATZ* gene structure, and batch-SMART analysis, the type and location of the conservative domain are presented here, with rectangles for exons, horizontal lines for introns, green for CDS regions, and other colors for corresponding conservative domains.

The MEME program identified 12 motifs for the 11 *GbPLATZ*s, named from motif 1 to motif 12 in ascending order of threshold value. Highly conserved motif distribution in each group ensures accurate gene classification and accurate regulation of downstream genes (Fu et al., 2020). The number and relative position of motifs among the members of the four groups were highly conserved, and there were both conserved and unique motifs among the four groups, highlighting the diversity of the *GbPLATZ* sequence structure (Figure 4A). All *GbPLATZ*s contained conservative motif 1/2/4. Motif 1 had the lowest threshold and represented a highly conserved domain. Two regions of the PLATZ-conserved structure, consisting of cysteine and histidine residues, are shown through different motifs. Motif 2/5/11/12 were all conserved regions composed of N-terminal cysteine and histidine residues. Motif 5 was replaced by motif 12 in group IV, consistent with *GbPLATZ1* and *GbPLATZ5*’s difference in domain1 cysteine from other members. Motif 11 was conserved in groups I and II. Motif 3 and motif 9 were regions of C-terminal cysteine residues in the PLATZ region, highlighting the differences between group IV and the other groups. Motif 4, located at the C-terminal of the amino acid sequence, was the translation termination region, and its conservatism might also be related to the nuclear localization signal. Motif 6 was located at the N-terminal of most members and may be involved in translation initiation. Motif 7 and motif-8 were specific to group II, and motif 10 was unique to group III.

According to gene structure (Figure 4B), all members contain 2–4 introns, except for the members of group IV that have no introns. The distribution and size of exons in the group members were relatively similar. All *GbPLATZ*s contained PLATZ-conserved domains in the central region, providing zinc-dependent DNA-binding capabilities. Both *GbPLATZ6* and *GbPLATZ11* in group III had a B-Box2 structure.

### Physicochemical Properties and Protein Structure of *GbPLATZ*s

Protein physicochemical analysis showed that the average protein length of the PLATZ TF in ginkgo was 270.27 AA, and *GbPLATZ1* was the longest at 348 AA. Molecular weights (MV) ranged from 25.84 to 38.74 kD, and the pI ranged from 8.02 to 9.26 (Table 2).

**Table 2:** Physicochemical properties of GbPLATZs protein.

Gene name	Protein length (aa)	Molecular weight (MV)kD	Theoretical pI	Instability index	Aliphatic index	Grand average of hydropathicity (GRAVY)	Predicted location(s)
*GbPLATZ1*	348	38.73813	9.22	57.87	74.8	−0.573	Nucleus
*GbPLATZ2*	251	27.92311	8.83	59.14	85.46	−0.233	Nucleus
*GbPLATZ3*	230	25.8386	8.83	60.43	78.39	−0.375	Nucleus
*GbPLATZ4*	257	28.79669	8.45	57.04	72.02	−0.400	Nucleus
*GbPLATZ5*	311	35.0471	9.12	51.51	66.78	−0.631	Chloroplast and Nucleus
*GbPLATZ6*	296	33.46763	8.02	50.16	62.94	−0.822	Nucleus
*GbPLATZ7*	250	28.31064	8.82	61.86	83.36	−0.262	Nucleus
*GbPLATZ8*	253	28.16948	9.24	70.99	82.85	−0.257	Nucleus
*GbPLATZ9*	258	29.08798	8.77	67.08	68.72	−0.537	Nucleus
*GbPLATZ10*	279	30.82318	8.43	49.37	74.77	−0.309	Nucleus
*GbPLATZ11*	240	27.36922	9.26	55.54	67.38	−0.647	Nucleus

**Figure 5:**
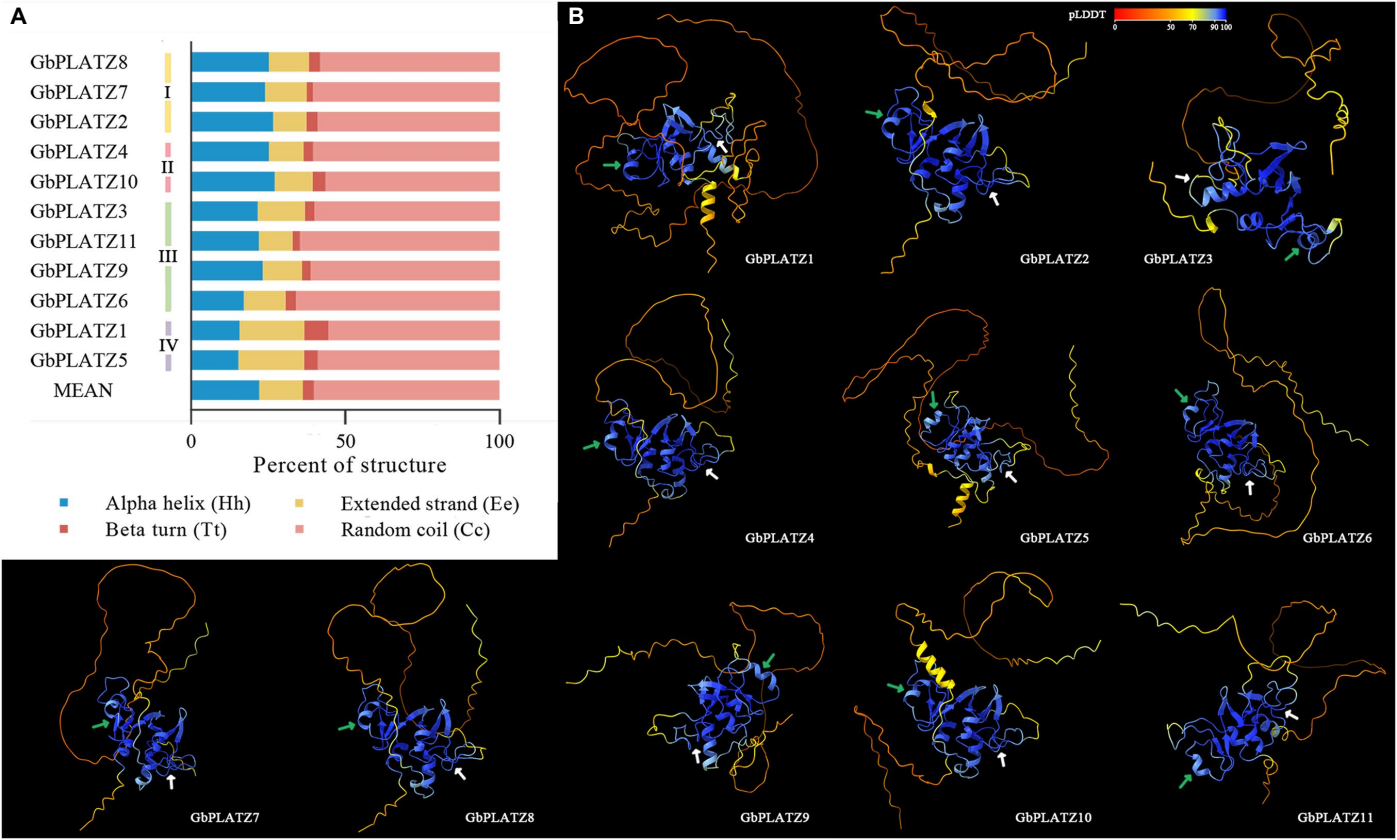
Protein secondary and tertiary structure prediction of GbPLATZs. **(A)** Prediction of protein secondary structure based on SOPMA. **(B)** Protein tertiary structure prediction based on AlphaFold2, protein coloring represents the pLDDT confidence measure, and green and white arrows represent the approximate location of domain 1 and domain 2, respectively.

The advanced protein structure was predicted using amino acid sequence data, and secondary structure characteristics maintained by hydrogen bonds were obtained by a self-optimized Prediction Method with Alignment (SOPMA). This method incorporates several independent secondary structure prediction methods to improve the accuracy of prediction. We found that the proportions of the four secondary structures of all GbPLATZ proteins were: random coil > α-helix > extended strand (β-sheet) > β-turn (Figure 5A). Group IV had more extended strands and β-turns, and fewer α-helixes than the other groups. There was no significant difference in the proportion of the four secondary structures in the other three groups, which was similar to the average. AlphaFold is a protein tertiary structure prediction algorithm with accuracy comparable to experimental verification using CryoEM, NMR or X-ray crystallography. We used AlphaFold2 to predict the tertiary structures of the PLATZ TFs of ginkgo. The tertiary structure is formed by further coiling and folding of proteins from the secondary structure. The tertiary structure was mainly maintained by the secondary bonds between the amino acid side chains, i.e., hydrophobic interactions, hydrogen bonds, Van der Waals forces and electrostatic interactions. In Figure 5B, the blue structures have >90% predictive confidence, and *GbPLATZ*s’ conserved PLATZ domain was included with a high degree of accuracy. There were more α-helixes and β-folds near domain 1. Domain 2 was dominated by random coils, and there was an α-helix near the C-terminal. The N-terminal of PLATZ domains in *GbPLATZ1* and *GbPLATZ5* and C-terminal in *GbPLATZ10* had one more α-helix than the other members, but the reliability of their tertiary structures was low, which may be related to unique functionality.

### Cis-Acting Element Analysis of *GbPLATZ*s

The PlantCARE (Lescot et al., 2002) and PLACE (Higo et al., 1998) databases were used to analyze cis-acting elements of a 2000 bp sequence upstream of *GbPLATZ*s according to the sequence similarity principle. PlantCARE contains sequence information for predicting plant cis-acting elements, enhancers and inhibitors, with a high reference value. Cis-acting elements predicted by PlantCARE can be divided into five categories: transcription-related, developmental, hormonal response, biological/abiotic stress, and others (Supplementary Table 3). Two cis-acting elements related to transcription, CAAT-box and TATA-box, were distributed in all upstream sequences of *GbPLATZ*s with a high number. In terms of type and quantity, the numbers of groups I and III had more developmental process-related cis-acting elements. For hormonal response, the highest number of related cis-acting elements were observed upstream of the group III members, particularly ABRE, methyl jasmonate (MeJA), CGTCA-box, and TCACG-motif, which might reflect the associated intragroup characteristics of group III. For stress response, all 11 *GbPLATZ*s contained the cis-acting element G-box, which is related to light response. The MYB and MYC binding sites were also common to all members. Our findings are similar to the characteristics of cis-acting elements of PLATZ TFs in other species. In addition, cis-acting elements for drought response, anaerobic response, low temperature response, defense, and stress response were present in the promoter regions of some members. The GCN4-motif and AACA-motif were two seed-specific cis-acting elements that appeared upstream of *GbPLATZ3/6/8* and *GbPLATZ9*, respectively. In addition, A/T-rich element was also found upstream of *GbPLATZ2*(I), *GbPLATZ3*(III), *GbPLATZ4*(II) and *GbPLATZ6*(III), which is probably related to the self-regulate of PLATZ.

**Figure 6:**
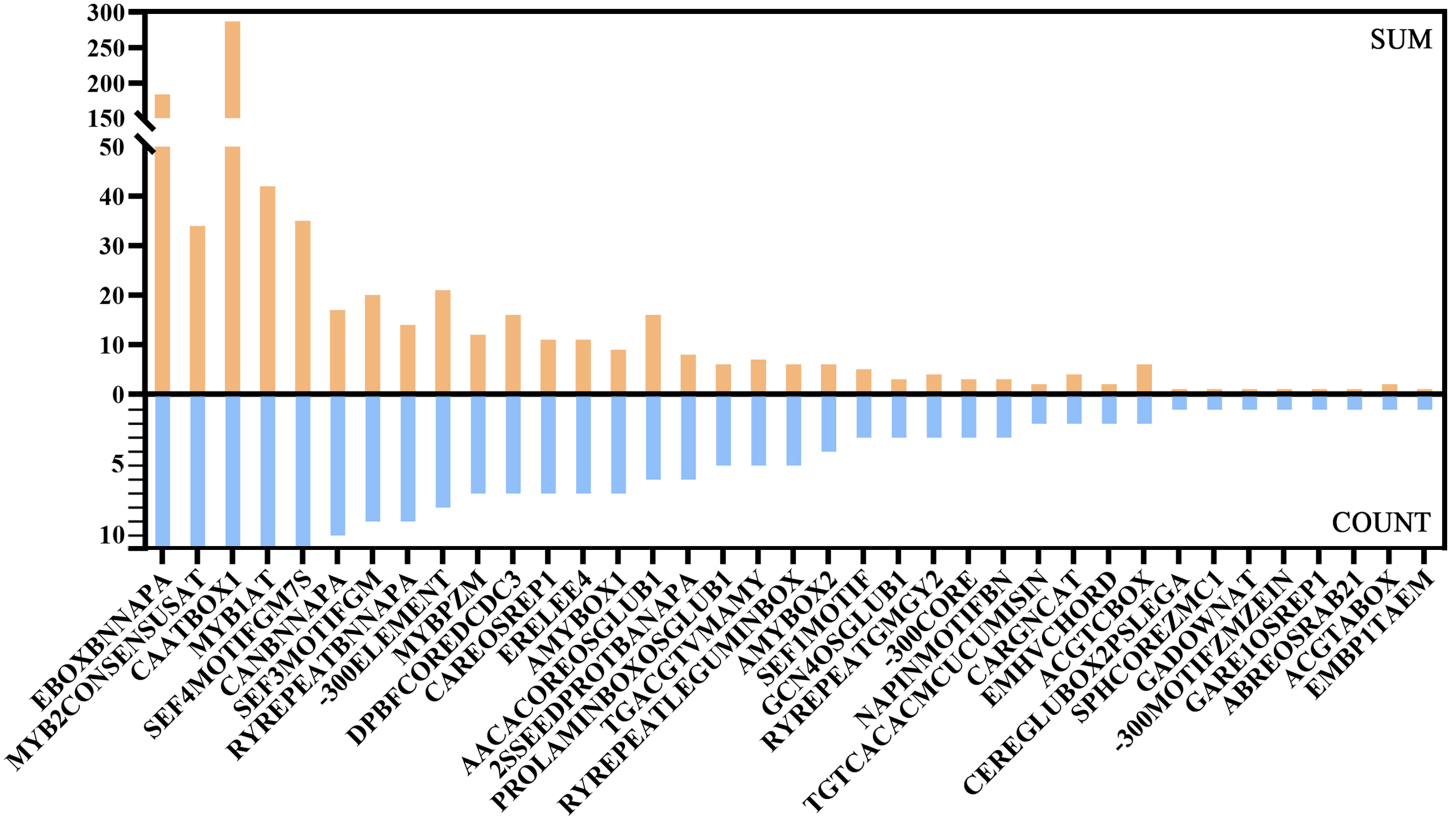
Cis-acting elements related to seeds from PLACE prediction results upstream of *GbPLATZ*s. SUM represents the total number of cis-acting elements upstream of the 11 *GbPLATZ*s. COUNT represents the number of *GbPLATZ*s whose upstream regions contain the respective elements.

The PLACE database consists of a sequence set of cis-acting elements verified by published experiments, with a smaller number of elements but more comprehensive and credible functional annotations. The obtained prediction results were analyzed according to the key words of functional annotation (Supplementary Table 4). Similar to the results predicted by PlantCARE, the five cis-acting elements EBOXBNNAPA, MYB2CONSENSUSAT, CAATBOX1, MYB1AT, and SEF4MOTIFGM7S upstream of the 11 *GbPLATZ*s were relatively abundant (Figure 6). All members had cis-acting components associated with seed storage proteins, including globulin, beta-conglycinin, rapeseed protein, and glutenin. In addition, all members except *GbPLATZ2* and *GbPLATZ8* contained the seed-specific RY-repeat element RYREPEATBNNAPA or RYREPEATLEGUMINBOX.

### Expression Pattern of *GbPLATZ*s

**Figure 7:**
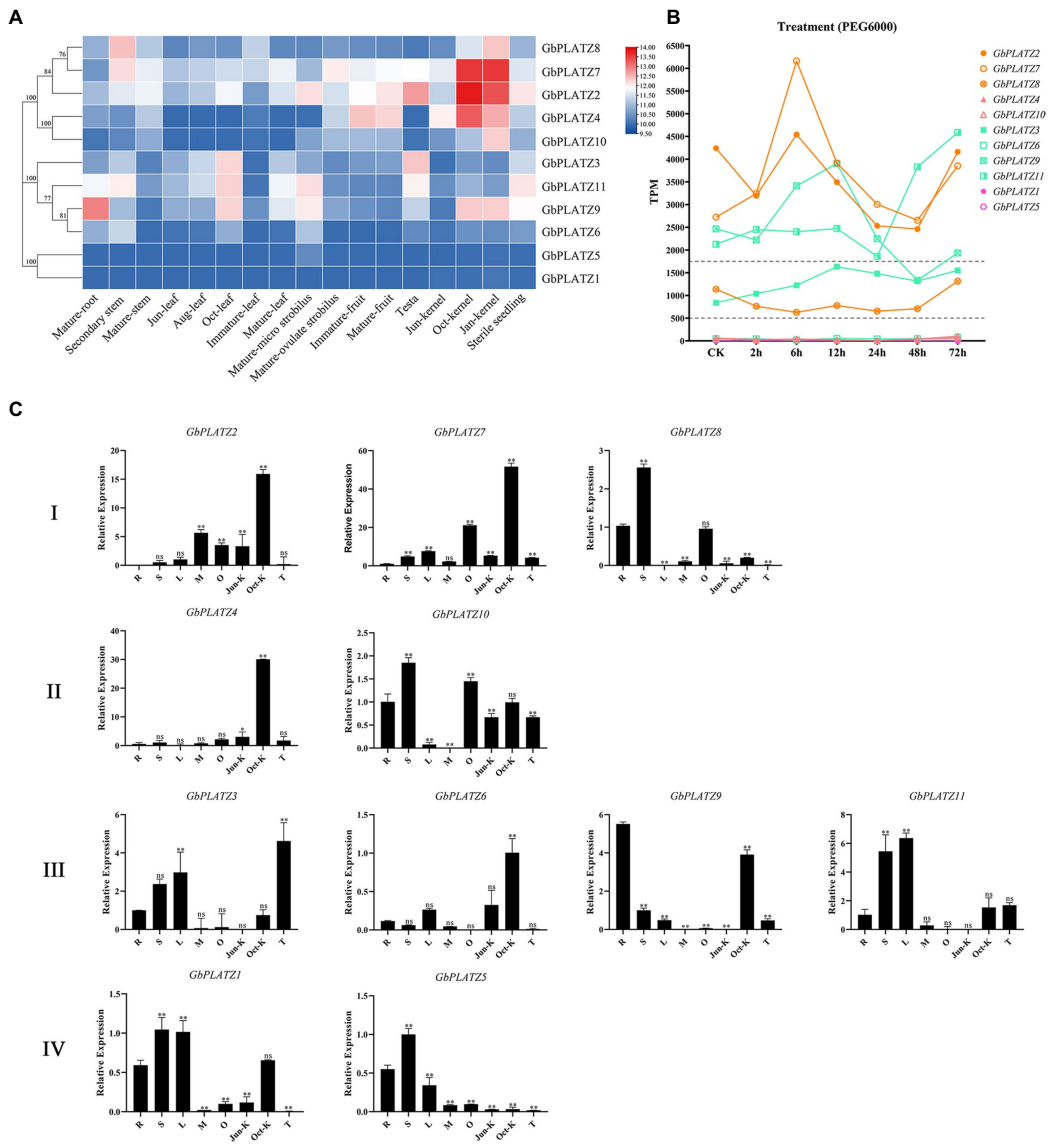
Expression pattern analysis of *GbPLATZ*s. **(A)** Expression of *GbPLATZ*s in different tissues based on NCBI data. TPM values calculated by READ_NUM were homogenized. The darker the red, the higher the expression level; the darker the blue, the lower the expression level. The evolution tree on the left of the heat map was drawn based on *GbPLATZ* gene sequence data and is consistent with Figure 2A. **(B)** The expression level of *GbPLATZ*s during PEG 6000 treatment based on NCBI data. The x-axis represents the time of PEG 6000 treatment, the y-axis represents the TPM value, and the dotted lines separate genes with distinct expression patterns. **(C)** Real-time quantitative data of each gene based on plant materials used in this study, the x-axis represents the different tissues. R, root; S, stem; L, leaf; M, micro-strobilus; O, ovulate-strobilus; Jun-K, kernel in June; Oct-K, kernel in October; and T, testa. F-test: ** < 0.01, * < 0.05.

According to RNA-seq data published by our laboratory and NCBI, we analyzed the expression profiles of *GbPLATZ*s in different tissues and developmental states as well as under drought stress (Figures 7A,B). Both the leaf and seed kernel included three development stages, and the fruit (here referring to the whole seeds of gymnosperms) included two sets of data with different maturity. The evolutionary tree on the left was constructed based on sequence similarity to compare the gene expression levels between tissues of members of different groups. The relative expression of *GbPLATZ9* was higher in vegetative roots, and that of *GbPLATZ7/8/11* was higher in stems, especially in secondary parts. *GbPLATZ3/6/9/11* in group III had high expression levels at the late stage of leaf development. In reproduction-related tissues, *GbPLATZ2/9/11* were highly expressed in the microstrobilus and *GbPLATZ2* was highly expressed in ovulate strobilus. Group I, group II, and *GbPLATZ9* of group III were mainly expressed in seeds. *GbPLATZ8* of group II and group I was only expressed in small amounts in the seed coat. The relative quantification of most genes by qRT-PCR was similar to the RNA-seq expression profile (Figure 7C). However, due to sampling differences and a batch effect in the public data, the quantitative results were inconsistent with the expression profile of transcriptome sequencing and require further analysis.

For abiotic stresses, our analysis used RNA-seq data from the NCBI database (accession: PRJNA604486) of ginkgo leaves simulating drought stress under PEG6000 treatment. The expression patterns of *GbPLATZ*s under drought stress can be divided into three categories (Figure 7B). All members of groups II and IV, as well as *GbPLATZ6* (group III), are located near the baseline, with little to no expression observed during drought stress. The expression amount of *GbPLATZ8* (group I) and *GbPLATZ3* (group III) were in the low level (500–2000) general, the expression level of *GbPLATZ8* decreased under drought stress, and increased only at around 72 h. The expression level of *GbPLATZ3* increased at the early stage of drought stress, and gradually stabilized at the late stage. *GbPLATZ2/7* (group I) and *GbPLATZ9/11* (group III) showed TPM values of >2000 TPM values in CK, and are members of the PLATZ TF family mainly expressed in ginkgo leaves. *GbPLATZ2/7/9* experienced peak expression levels within 12 h of drought stress and decreased with time, which may be related to the short-term response to stress. *GbPLATZ2* and *GbPLATZ9* recovered to almost the same level after 72 h, and the expression of *GbPLATZ7* increased after 48 h. The expression of *GbPLATZ11* did not change much in the early stage of drought treatment, but increased rapidly after 24 h. *GbPLATZ11* and *GbPLATZ7* may play an important role in the late response to drought stress.

### Subcellular Localization of *GbPLATZ* Proteins

**Figure 8:**
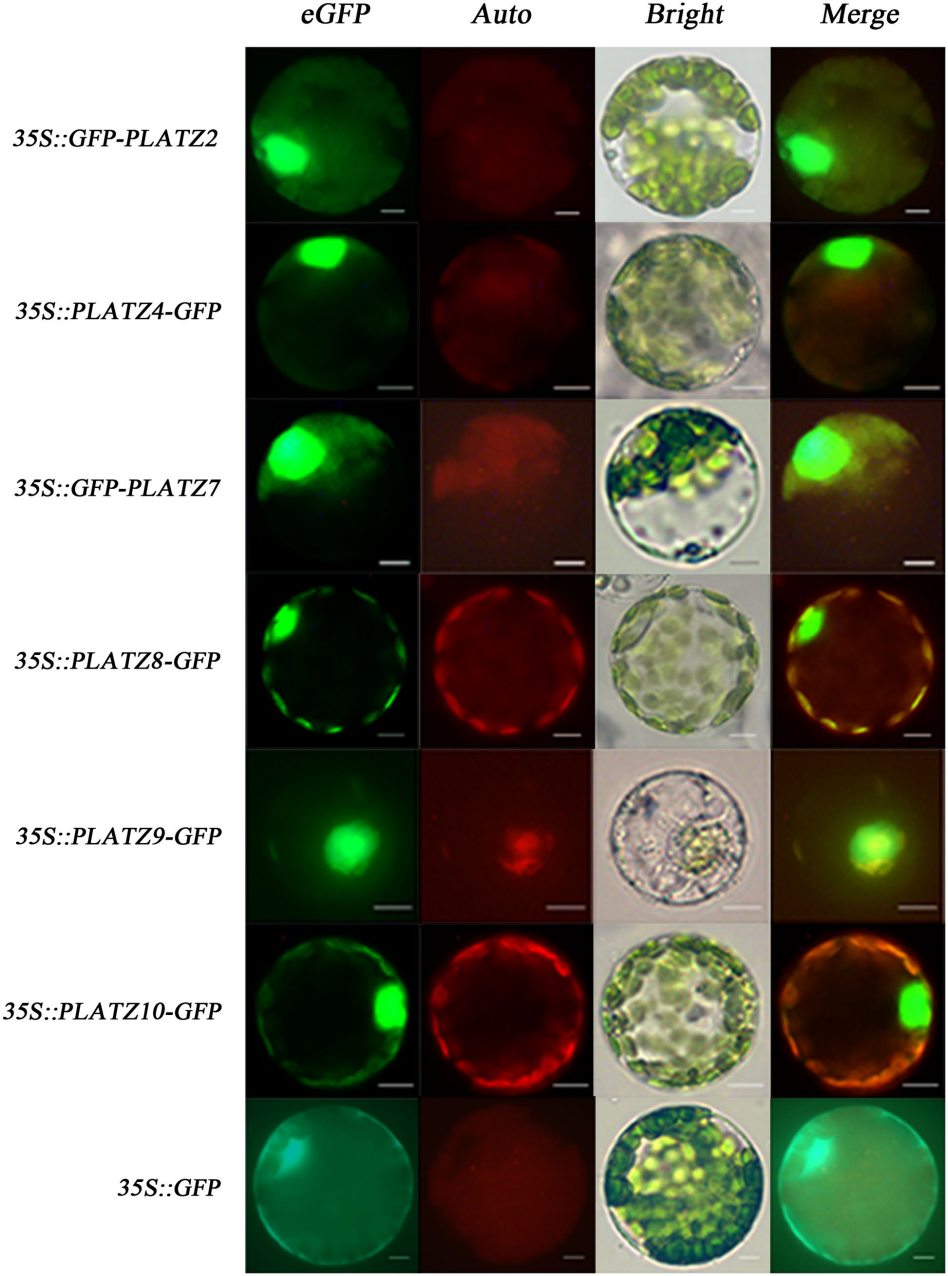
The subcellular localization of GbPLATZ proteins in ginkgo. The fusion location of the GFP gene sequence is shown on the left. Scale bars = 10 μm.

Only *GbPLATZ5* was predicted to be double-localized in the chloroplast and nucleus, while the other 10 *GbPLATZ*s were nuclear-localized (Table 2). The prediction and corresponding threshold of the nuclear localization sequence are shown in Supplementary Table 5. We selected six GbPLATZ proteins with high expression levels in the seed, especially the kernel, for transient expression to verify the accuracy of the subcellular localization predictions. For *GbPLATZ2/4/7/8/9/10*, a green fluorescent protein GFP sequence (constituting the fusion gene) was connected to the C- or N-terminal, which was driven by the constitutive promoter CaMV35S and expressed instantaneously in ginkgo mesophyll protoplasts. Unfused *35S::GFP* was used as a control group. All fusion proteins were located in the nucleus, but the control GFP protein was distributed at the subcellular level, including in the nucleus, cytoplasm, and organelles (Figure 8). These results suggest that the proteins encoded by the *GbPLATZ*s, like most TFs, are located in the nucleus and might function as general TFs participating in transcriptional level activities.

## Discussion

A total of 11 PLATZ TFs were identified in *G. biloba*. Although ginkgo has a relatively large genome (9.87Gb), the number of PLATZ TF family members was similar to that of *A. thaliana* (12), but less than that of rice (15), common wheat (*Triticum aestivum*, 46; He et al., 2021), maize (17; Wang et al., 2018), and Chinese cabbage (*Brassica Rapa*, 24; Azim et al., 2020), among others. Tandem repeat analysis of PLATZ genes in wheat and cabbage showed that gene replication events increased the number of members associated with the protein family. Compared with gymnosperms, the genome-wide replication event (WGD) may be the main evolutionary driver of the rapid expansion of angiosperms since the mid-Cretaceous (Niu et al., 2021), which corresponded with an increase in the number of protein family members. In gymnosperms, studies have shown that expansion of the ginkgo genome, accompanied by a notable extension of introns, was mainly caused by the insertion of long terminal repeats rather than whole-genome duplication events. This is consistent with the small number of PLATZ protein family members and the absence of collinearity observed in the present study.

The zinc-finger protein domain, i.e., the conserved region of PLATZ TFs, is one of the most common structures of cellular proteins and mediates the interaction between proteins and other biomolecules, including DNA and proteins (Azim et al., 2020). The *GbPLATZ*s all had two distant conserved zinc-finger regions, B-box and PLATZ. The N-terminal region conserved domain (B-box) was slightly different between the two members of group IV, and the last conserved cysteine (C) was replaced by glycine (G), which may have a great influence on the function of this group. Considering its degeneracy, we further analyzed the amino acid codon. The glycine of *GbPLATZ1/5* was “GGA,” corresponding to “TGC” (*GbPLATZ4/6/7/8*) and “TGT” (*GbPLATZ2/3/9/10/11*) in the other members at the cysteine position of the codon. The non-conserved condition of a single amino acid in the group IV conserved domain was caused by at least two nucleotide changes, with a T-G transversion in the first position and an A-T transformation or A-C transversion in the third position. Given the evolutionary relationship of the 11 PLATZ proteins in ginkgo, group IV was independent of the other groups, and thus so the changes in this region could not be predicted. Combined with the expression levels of these genes (Figure 7A), the two members of group IV were likely to change the binding ability of the zinc finger due to amino acid changes in this conserved domain, which could eventually lead to changes in function.

Phylogenetic analysis of the *GbPLATZ*s showed that the 11 members could be divided into four distinct groups, with groups I and II being closely related. In the multi-species analysis of maize, *A. thaliana*, Norway spruce, and ginkgo PLATZs, the four groups of *GbPLATZ*s occurred in different branches and were more closely related to the PLATZ genes of the Norway spruce. This suggests that PLATZ genes are highly conserved in seed plants and originate from a common ancestor; thus, PLATZ genes may play an important evolutionary role in plants. However, in the phylogenetic tree constructed from these four species, angiosperms had newly derived branches, which reflects the differences in the evolution of seed plants. Combined with the phylogenetic tree of seven species (Supplementary Figure 2), soybean and rice were found also to have many members in the new group derived from angiosperms. Nevertheless, group IV maintained its unique phylogenetic performance, with only one loblolly pine and Arabidopsis gene in the same branch. The loblolly pine gene contains only one intron of about 100 bp, with a similar gene structure to ginkgo, and the Arabidopsis gene had a low bootstrap value which diminishes its relevance.

In the analysis of *GbPLATZ* motifs and structure, the within-group similarity of modules was very high. Except for the modules involving conserved regions and translation initiation/termination regions, the four groups of *GbPLATZ*s had a diversity of conserved motifs, with motif 11 being unique to groups I and II, motif 7/8 unique to group II, motif 10 specific to group III, and motif 9/12 specific to group IV. All of the motifs showed differences in sequence characteristics at the group level, which represents the basis of functional differentiation. Similarly, the exon-intron structural characteristics of these *GbPLATZ*s differed between groups, which indicates a degree of structural variation within the gene family. In contrast to the other species analyzed here, neither of the members in group IV contained introns. Large genes with long introns are highly expressed, and DNA methylation may be involved in the accurate identification of exons of ultra-long introns (Niu et al., 2021). In contrast, intron-free gene expression levels in Chinese pine were low, consistent with the group IV members of the PLATZ family in ginkgo. Combined with the observed variation in the conserved domain, the *GbPLATZ*s in group IV differed from other genes at the DNA level, which may indicate the downstream expression of genes specific to this group. This was partly confirmed by the prediction results of higher protein structure.

After receiving signals from the cell membrane, TFs synthesized in the cytoplasm are transferred to the nucleus and combined with cis-acting elements of downstream regulatory genes, and in this way, TFs play a regulatory role at the transcriptional level (Liu et al., 2018). We also observed several cis-acting elements in the promoter region upstream of the *GbPLATZ*s. Of the four groups, group I and group III had more development-related elements, and group III also contained a variety of hormone response elements, indicating that members of these two groups may participate specifically in growth and development. Multiple stress-responsive elements were present in the promoter regions of some family members, similar to the characteristics of cis-acting elements of PLATZ genes in other species. As protein, the synthesis of TF itself is also regulated at the transcription level. There have been many reports on the self-regulation of TFs. For example, ABI3 and FUS3 in LAFL (the core regulatory network of seed development: LEC1-ABI3-FUS3-LEC2) have the ability of self-regulation (Santos-Mendoza et al., 2008). In the prediction of this study, four *GbPLATZ*s upstream within 2000 bp contain A/T-rich elements, which may enable these members to self-regulate. From the PLACE database, we found that all upstream *GbPLATZ*s had cis-acting elements related to seed development. Along with other well-established functions, our findings suggest that the PLATZ TF family plays an important and conserved role in seed development.

*GbPLATZ* expression differed significantly between tissues, indicating that the family may have a variety of functions. Indeed, *AtPLATZ1* (Dong et al., 2021) induces expression of ABA in roots and controls the growth of taproots; *AtPLATZ7* (Yamada et al., 2020) controls the size of the root meristem through ROS signals; *AtPLATZ3* (Jun et al., 2020) promotes leaf growth by promoting the rate and duration of cell proliferation in leaves. In seeds, *SG6/GL6* (Wang et al., 2019; Zhou and Xue, 2020) of rice and *ZmPLATZ2/12* (Li et al., 2017, 2021) of maize have also been reported to play a regulatory role. In ginkgo, *GbPLATZ9* was highly expressed in roots and seed kernels at the later stage of seed development. Considering that embryogenesis in ginkgo at this stage includes root genesis, his gene may have a potentially important function in roots. Similarly, the members of group I, especially *GbPLATZ8*, were highly expressed in stems, and may be related to stem development; however, a more detailed evaluation of ginkgo genes related to stem development is required. According to the RNA-seq data, group III played an important role in leaf development, especially in the later stage of development. In reproductive tissues of ginkgo, the gene expression levels of male and female flowers differed markedly between RNA-seq and qRT-PCR data. For qRT-PCR, the flower buds used for RNA extraction are morphologically formed by new leaf buds wrapped around immature megasporophytes/microsporophytes. Therefore, the qRT-PCR results were not completely derived from reproductive organs and could not represent the specific gene expression in reproductive tissues. RNA-seq data showed that group II was not involved in the development of ovulate strobilus or microstrobilus, *GbPLATZ2/8* of group I and *GbPLATZ9/11* of group III were associated with reproduction-related tissues. In seeds, the *GbPLATZ*s except for groups I, II, and III were related to seed development. In the kernel, the members of groups I, II, and *GbPLATZ9* of group III played an important role. In the seed coat, *GbPLATZ2/7* of group I and *GbPLATZ3/9/11* of group III played a major role, while the members of group II showed almost no expression. The expression of group IV in each tissue was relatively low, similar to some PLATZ members in maize, wheat, Chinese cabbage, and other species. Based on the above expression patterns verified with different techniques and data sources, group I of ginkgo PLATZ TFs was expressed in all tissues, with especially high expressed in seeds; the expression of group II was specific to seeds, especially kernels; group III was mainly expressed in nutritional tissues; and the expression of group IV was inconsistent between tissues.

## Conclusion

A total of 11 *GbPLATZ*s distributed on six chromosomes were identified from the 9.87GB ginkgo genome, which was similar to the number of the model plant *A. thaliana* and less than that of angiosperms such as maize, soybean, and rice. There was no collinearity or tandem repetition in these genes, which was consistent with the fact that gymnosperms were relatively primitive in evolution and had few genome-wide replication events. Phylogenetic analysis showed that *GbPLATZ*s could be divided into four groups, and new groups might be derived from angiosperms. The analysis of the conservative domains and sequence motifs showed that the sequence characteristics of each group were evident, and members of group IV were significantly different from others, accompanied by an amino acid change in the conservative domain and lack of introns. Based on the comparison results of the cis-acting element database, *GbPLATZ* genes may be affected by a variety of hormone and environmental factors, and there were a variety of response elements related to seed development. Based on sequencing data and qRT-PCR tissue-specific expression results, most PLATZ genes of ginkgo played a regulatory role in seed growth and development, similar to those of angiosperms previously studied. RNA-seq data from the public database and our laboratory also reflected the strong response of some members to stress, suggesting that some genes also play a role in abiotic stress response. This study provided new insights into the conservation and evolution of PLATZ transcription factors in seed plants and provides valuable information for further studies on the regulatory functions of *GbPLATZ*s in ginkgo seed growth and development and stress tolerance.

## Data Availability Statement

The datasets presented in this study can be found in online repositories. The names of the repository/repositories and accession number(s) can be found in the article/supplementary material.

## Author Contributions

LX and MX conceived and designed the project. XH and YT undertook the molecular biology experiment. XH and HR participated in the data analysis. YQ provided the scripts for analysis. XH drafted the manuscript. LX, HR, and MX modified the manuscript. All authors contributed to the article and approved the submitted version.

## Funding

This research was funded by the National Natural Science Foundation of China (31971689).

## Conflict of Interest

The authors declare that the research was conducted in the absence of any commercial or financial relationships that could be construed as a potential conflict of interest.

## Publisher’s Note

All claims expressed in this article are solely those of the authors and do not necessarily represent those of their affiliated organizations, or those of the publisher, the editors and the reviewers. Any product that may be evaluated in this article, or claim that may be made by its manufacturer, is not guaranteed or endorsed by the publisher.

## Footnotes

^1^ http://pfam.xfam.org/family/PF04640

^2^ https://web.expasy.org/protparam/

^3^ http://www.premierbiosoft.com/molecular_beacons/index.html

## Supplementary Material

Supplementary Figure 1 Comparison of the 11 GbPLATZs amino acid sequences. The deeper shading of the letters representing amino acids indicates that the site is more conservative. The last line is the conserved domains of PLATZ TF family, and the other domains were underlined in different colors.

Supplementary Figure 2 Comparison of PLATZ sequences in Arabidopsis thaliana (AT), maize (Zm), rice (Os), soybean (Glyma), loblolly pine (PITA), Norway spruce (MA), and *G. biloba* (Gb). The branch of the ginkgo gene is shown in red. The size of the point on the branch indicates the self-spreading value of the corresponding branch. The branch length is proportional to the rate of amino acid change.

Supplementary File The sequences of the 11 GbPLATZ proteins.
