# Supplementary figures and images for: Genome-Wide Identification of PLATZ Transcription Factors in Ginkgo biloba L. and Their Expression Characteristics During Seed Development
Source: Front Plant Sci. 2022 Jun 23;13:946194. doi: 10.3389/fpls.2022.946194 (PMC9262033; doi:10.3389/fpls.2022.946194)

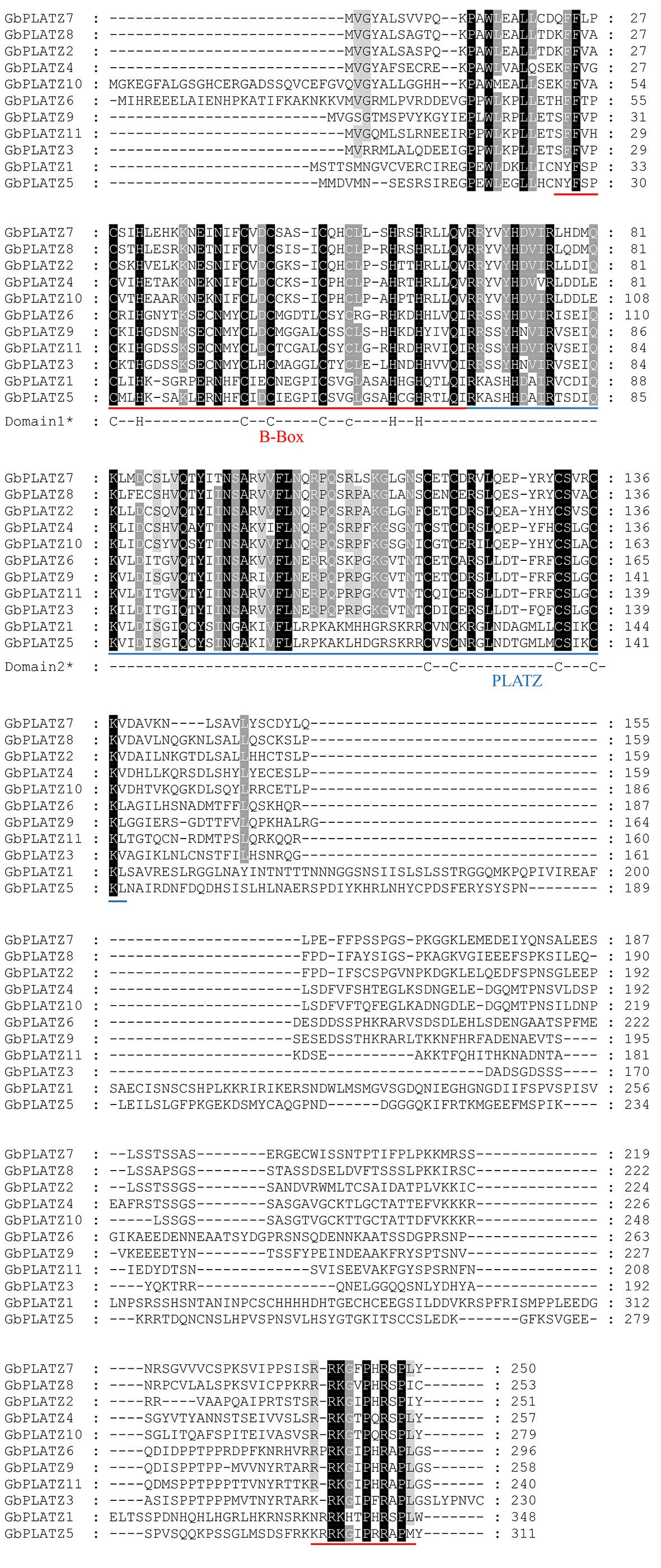

Supplement: Supplementary Figure 1 — Comparison of the 11 GbPLATZs amino acid sequences. The deeper shading of the letters representing amino acids indicates that the site is more conservative. The last line is the conserved domains of PLATZ TF family, and the other domains were underlined in different colors. [file Image_1.tif]

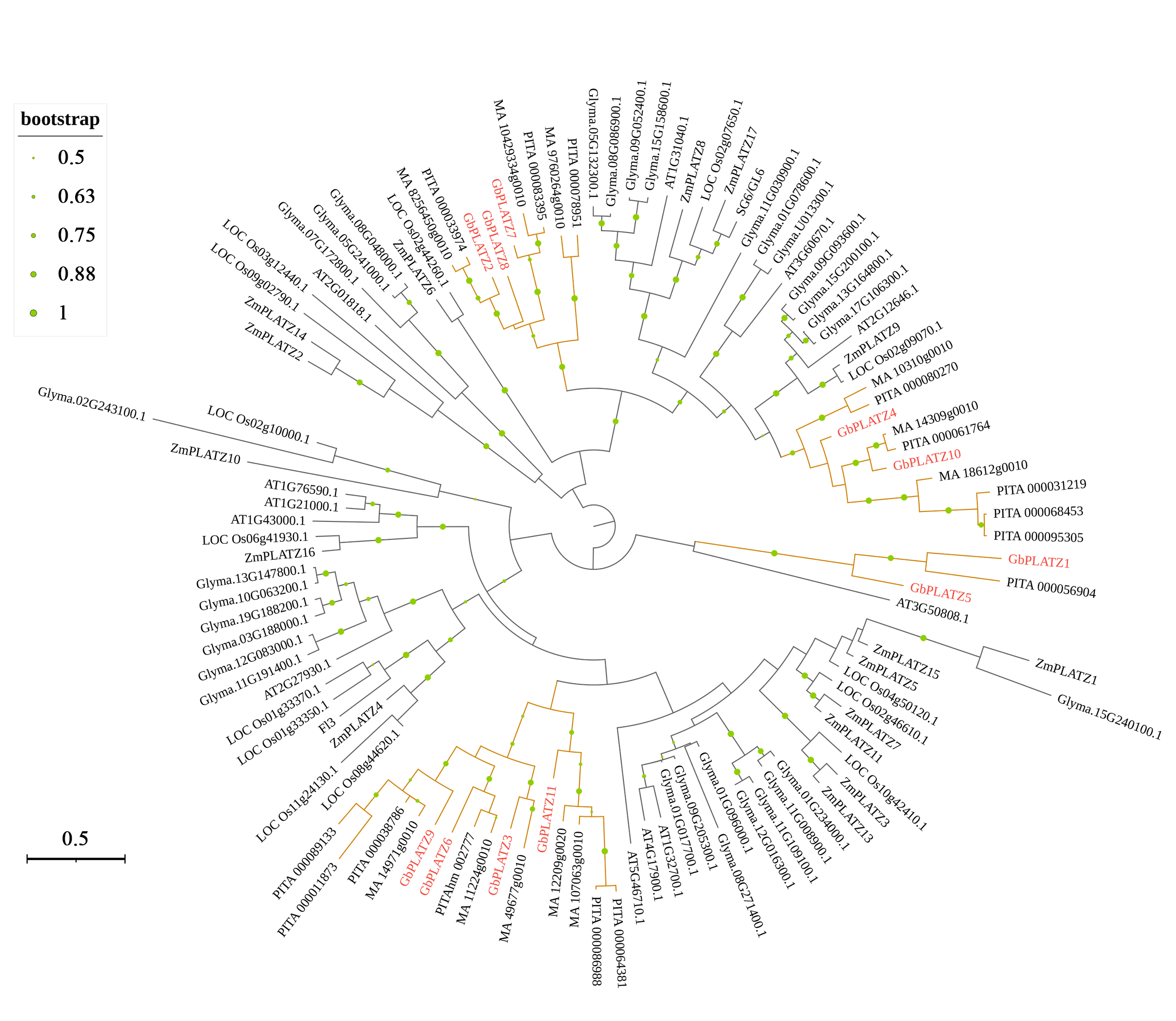

Supplement: Supplementary Figure 2 — Comparison of PLATZ sequences in Arabidopsis thaliana (AT), maize (Zm), rice (Os), soybean (Glyma), loblolly pine (PITA), Norway spruce (MA), and G. biloba (Gb). The branch of the ginkgo gene is shown in red. The size of the point on the branch indicates the self-spreading value of the corresponding branch. The branch length is proportional to the rate of amino acid change. [file Image_2.tif]
